# Acute pyelonephritis revealing an exceptional form of horseshoe kidney: A case report

**DOI:** 10.1016/j.ijscr.2023.108607

**Published:** 2023-08-02

**Authors:** Ahmed Jdaini, Anouar El Moudane, Hammou El Farhaoui, Youssef Kouiss, Anass El Alaoui, Ali Barki

**Affiliations:** Department of Urology, Mohammed VI University Medical Center, Mohammed The First University Oujda, Morocco

**Keywords:** Horseshoe kidneys, Fibrous isthmus, Metanephric, Renal fusion, Case report

## Abstract

**Introduction:**

The horseshoe kidney is a malformation of the upper urinary tract where the kidney is U-shaped. This condition results the fusion of the lower poles of the right and left kidneys on the midline.

**Case presentation:**

We report a case of pyelonephritis revealing a rare form of horseshoe kidney with fusion of the lower pole of the right kidney with the upper pole of the left kidney, initially treated with urine drainage.

**Discussion:**

Multiple etiological conditions may contribute to the development of a horseshoe kidney, in particular: the genetic/chromosomal predisposition, intrauterine environment and structural factors affecting kidney development and migration.

**Conclusion:**

Horseshoe kidneys reveal a veritable range of anatomical and embryological peculiarities, always suspect pyelonephritis on a horseshoe kidney in the presence of febrile abdominal pain in a patient with malformations.

## Highlights

• The horseshoe kidney is a malformation of the upper urinary tract where the kidney is U-shaped , this condition results the fusion of the lower poles of the right and left kidneys on the midline.
• We report a case of pyelonephritis revealing a rare form of horseshoe kidney with fusion of the lower pole of the right kidney with the upper pole of the left kidney
• Generally, renal fusion anomalies occur between 4 and 6 weeks of development. There are several hypotheses regarding the cause of horseshoe-shaped kidneys, including positional factors, , intrauterine factors such as maternal environment , metanephric cell migration disorders and exposure to teratogens, as well as associated genetic factors and chromosomal abnormalities

## 1 Introduction

Horseshoe kidney is a renal fusion disorder. It is usually more common in males, with a prevalence of 1 in 400 births. In this disorder, the kidneys are connected by a parenchymal or fibrous isthmus that traverses the midline. Embryologically, the anomaly appears between the fourth and sixth weeks of gestation when the metanephric part of the blastema fuses before the ascension and rotation of the kidneys. Horseshoe kidney is generally associated with an increased risk of infection, obstruction, stone formation, and kidney tumors [1]. We report a case of pyelonephritis revealing a rare form of horseshoe kidney with fusion of the lower pole of the right kidney with the upper pole of the left kidney. The work has been reported in line with the SCARE 2020 criteria [6].

## 2 Case report

**Fig. 1:**
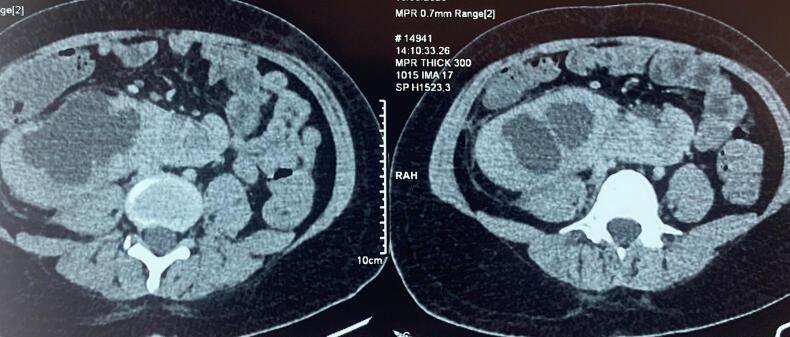
Scan image showing fusion of the lower pole of the right kidney with the upper pole of the left kidney and right renal dilatation.

**Fig. 2:**
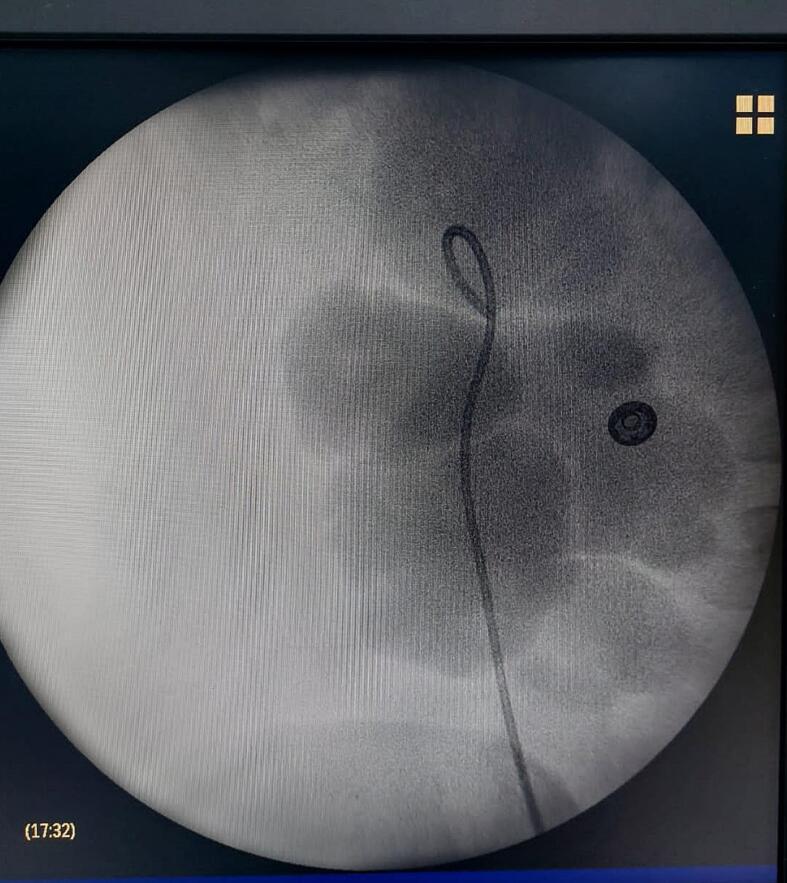
Scopic image showing right ureter hydronephrosis with double-J catheterization

A 30-year-old man, with a history of mental handicap and epilepsy under treatment, operated on in the past for hypospadias presented in emergency with acute abdominal pain and fever. Clinical examination revealed an abdominal tenderness and rigidity associated with a fever at 39°. Urine culture was positive, with the presence of a germ on direct examination, the abdomino-pelvic CT scan showed a fusion of the lower right and upper left poles, with dysrotation of the right kidney and pyelo-caliceal dilatation with pyelon measured at 49 mm with possible junction syndrome (Fig. 1). The patient was admitted to a urology unit and treated with antibiotics based on 3rd generation cephalosporin and aminoglycoside, and was promptly transferred to the operating room, where he underwent a right double-J catheterization under general anesthesia (Fig. 2). The antibiotic was continued according to the antibiogram, the evolution was marked by the elimination of fever and pain. The patient returned home under antibiotic treatment, patient reports disappearance of pain.

## 3 Discussion

The horseshoe kidney is a fusion anomaly of the kidney. Multiple etiological conditions may contribute to the development of a horseshoe kidney, in particular: the genetic/chromosomal predisposition, intrauterine environment and structural factors affecting kidney development and migration [2]. Embryologically, each kidney is composed of two distinct cell populations: the ureteral bud and the metanephric blastema, the collecting system is formed by the ureteral bud, while the functional kidney is formed by the metanephric blastema. These two elements are combined in the sacred region.

Generally, renal fusion anomalies occur between 4 and 6 weeks of development. There are several hypotheses regarding the cause of horseshoe-shaped kidneys, including positional factors, intrauterine factors such as maternal environment, metanephric cell migration disorders and exposure to teratogens, as well as associated genetic factors and chromosomal abnormalities [2].

In weeks 6 to 8 of development, renal ascension is coupled with 90° medial rotation [3]. As a result, the hilum moves from anterior to medial position, and an inclination of the renal axis. The spectre of horseshoe kidney disorders includes malrotation. The most frequent cases are incomplete rotation or non-rotation. However, hyper-rotation and reverse rotation can also occur [3].

Fusion disorders can be caused by irregular fluctuations in growth and ventral flexion of the caudal fetus inside a veritable confined basin.

There is no genetic cause for horseshoe kidney in humans. But a number of regulatory steps in kidney development have not been entirely elucidated, and could contribute to a better understanding of etiology [4]. The predominance of men is well described, family cases between father and son have also been reported, and monozygotic twins. This is a strong argument that the genetic programming of the fetus can influence the development of the child.

Some authors suggest that urological malformations associated with chromosomal disorders are in part a consequence of retarded consequence of development of the nephrogenic blastema and the ureteral bud [5].

In Edwards syndrome, horseshoe-shaped kidneys appear in two-thirds of patients [7], and in Downs syndrome, the occurrence of horseshoe kidneys is probably <1 % [8]. In Turner syndrome, horseshoe kidneys are present in 14 % to 20 % of patients [9], with a lower incidence of renal malformations in people with mosaicism.

The maturing kidney gains and loses several sources of vascular supply during ascent. In the pelvis, the median sacral artery and the internal and external iliac vessels nourish it. Subsequently, it may be fed directly by the aorta or by a branch of the aorta.

In our case, the horseshoe kidney was associated with other anomalies such as hypospadias and mental retardation, the acute abdominal pain revealed an exceptional form of horseshoe kidney in which the lower pole of the right kidney is fused with the upper pole of the left kidney, generally the two lower poles are fused together.

## 4 Conclusion

Horseshoe kidneys reveal a veritable range of anatomical and embryological peculiarities. Various mechanical and genetic associations have been suggested. Their relative migration is incomplete, resulting in a final position ranging from the normal renal fossa to the pelvis, always suspect pyelonephritis on a horseshoe kidney in the presence of febrile abdominal pain in a patient with malformations.

## Ethical approval

The ethical approval has been exempted by our institution.

The patient gave written permission to publish his case findings before he passed away.

## Funding

No.

## CRediT authorship contribution statement

Ahmed Jdaini, Anouar El moudane, Hammou El farhaoui, Anass El Alaoui: write the paper.

Ali Barki supervised the paper writing.

## Guarantor

Ahmed Jdaini

## Consent

Written informed consent was obtained from the patient for publication of this case report and accompanying images. A copy of the written consent is available for review by the Editor-in-chief of this journal on request.

## Declaration of competing interest

The authors declare that there is no conflict of interests regarding the publication of this article
